# Medico-legal case series of litigation involving chronic post-herniorrhaphy inguinal pain: insights from Italian civil verdicts

**DOI:** 10.1007/s10029-025-03472-y

**Published:** 2025-10-08

**Authors:** Roberto Cirocchi, Luca Tomassini, Salvatore Guarino, Andrea Mingoli, Diletta Cassini, Bruno Cirillo, Massimo Lancia, Piergiorgio Fedeli, Paolo Bruzzone

**Affiliations:** 1 Department of Digestive and Emergency Surgery, “S.Maria” Hospital, Terni, Italy; 2 Department of Medicine and Surgery, University of Perugia, Perugia, Italy; 3 School of Advanced Studies, University of Camerino, Camerino, Italy; 4 Department of General Surgery - IRCCS Multimedica, Milan, Italy; 5 Department of Surgery, Sapienza University of Rome, Rome, Italy; 6 ASST Nord Milano-Department of General and Rery, Sesto San Giovanni Hospital, Sesto San Giovanni, Italy; 7 School of Law, Legal Medicine, University of Camerino, Camerino, Italy; 8 Department of General and Specialist Surgery, University of Roma La Sapienza, Rome, Italy

**Keywords:** Chronic pain, Inguinal hernia, CPIP, Medico-legal claims, Nerve injury, Malpractice

## Abstract

**Purpose:**

Chronic postherniorrhaphy inguinal pain (CPIP) is a recognized postoperative complication and a potential trigger for malpractice litigation. This case series presents a descriptive medicolegal analysis of civil verdicts involving CPIP following inguinal hernia repair in Italy.

**Methods:**

A retrospective review was performed using the Italian Ministry of Justice’s *Banca Dati di Merito*, examining malpractice verdicts from 2015 to 2025 related to CPIP after hernioplasty. Seventeen cases met the inclusion criteria. Variables collected included type of nerve injury, surgical technique, fixation method, informed consent, reinterventions, and symptom profile. Data were summarized using descriptive statistics.

**Results:**

Compensation was awarded in 4 of 17 cases (23.5%). Nerve injury was documented in 13 cases (76.5%), with genitofemoral nerve involvement in 6 of these (46.2%). Among the six cases with genitofemoral injury, four received compensation (66.7%), while no compensation occurred in cases without such injury. Neurectomy was performed in 5 cases, three of which resulted in compensation (60.0%) compared with 1 of the 12 cases without neurectomy (8.3%). Informed consent forms were available in 5 cases; in three of these, the risk of nerve injury was omitted, with one case leading to compensation.

**Conclusion:**

This descriptive case series highlights a potential association between genitofemoral nerve injury and compensation outcomes in CPIP-related litigation. In most cases involving genitofemoral injury, compensation was awarded. Neurectomy also appeared to show a trend toward legal relevance. These findings reinforce the medico-legal importance of accurate nerve identification, thorough intraoperative documentation, and attentive postoperative management in hernia surgery. Further prospective studies are warranted to confirm these preliminary observations and inform both surgical and legal best practices.

## Preface

Chronic post-herniorrhaphy inguinal pain (CPIP) is a recognized postoperative complication and a growing concern in surgical and medico-legal settings [1, 2]. Defined by the International Association for the Study of Pain (IASP) as discomfort persisting beyond three months after hernia repair, CPIP may impair mobility, limit employability, and negatively affect patients’ quality of life [3].

The condition is multifactorial in origin. Somatic pain—by far the most common form—stems from mesh tension, fibrosis, or scar tissue and is often localized [3, 4]. Neuropathic pain, far less frequent (< 1% of postoperative inguinodynias) [1, 5, 6], arises from direct injury or entrapment of nerves such as the ilioinguinal, iliohypogastric, or genitofemoral, typically manifesting as burning or shooting pain [7, 8]. Differentiating between these etiologies is essential for effective treatment and appropriate legal assessment and may influence litigation outcomes.

Reported CPIP prevalence varies due to inconsistent definitions and study designs, but recent estimates place the global incidence at 17.0% (95% CI: 12.8–21.7%)—with higher rates observed in Europe (18.7%) than in Asia (14.7%) or North America (6.0%) [2]. Identified risk factors include younger age, female sex, preoperative pain, and a history of recurrent hernia repair [2, 9, 10].

While clinical awareness of CPIP is increasing, its medico-legal implications remain under-investigated, particularly regarding documentation quality, informed consent, and causality of nerve injury. In Italy, no previous studies have examined how these elements feature in malpractice litigation. Given the limited sample size and the descriptive nature of the data, this study is best classified as a retrospective case series. It aims to help fill this gap by analyzing civil rulings involving CPIP following hernioplasty, with attention to compensation patterns and the procedural features described in judicial outcomes. The study provides a descriptive overview intended to inform future research on the intersection of surgical practice and medico-legal accountability.

## Materials and methods

This retrospective case series was designed as an observational medicolegal investigation based on published civil litigation records involving chronic postherniorrhaphy inguinal pain (CPIP) following inguinal hernia repair. All cases were independently reviewed by two assessors (R.C., L.T.) using a standardized data extraction protocol. Discrepancies in classification or interpretation were resolved by consensus, or, when necessary, with input from a third reviewer (P.B.).

The dataset was compiled by retrieving legal cases from the *Banca Dati di Merito*, a publicly accessible online database managed by the Italian Ministry of Justice. This archive contains anonymized summaries of civil and criminal court rulings issued nationwide. Sources included judgments from both the *Procura della Repubblica* (Public Prosecutor’s Office), which handles firstinstance legal actions, and the *Corte d’Appello* (Court of Appeal), which adjudicates civil appeals. The search covered rulings published between 2015 and 2025, using Italianlanguage terms pertinent to the clinical and legal aspects of CPIP, including: *dolore inguinale cronico* (chronic inguinal pain), *ernia inguinale* (inguinal hernia), *neuralgia postoperatoria* (postoperative neuralgia), *contusione nervosa* (nerve injury), and *risarcimento danni* (damage compensation). Terms were selected to maximize sensitivity in identifying relevant malpractice cases. Ethical approval was obtained from the Bioethical Committee of the University of Perugia (Protocol No. 169168, 12 May 2025). As the study used only publicly available, anonymized records, patient consent was waived in accordance with national guidelines and institutional policy.

**Fig. 1:**
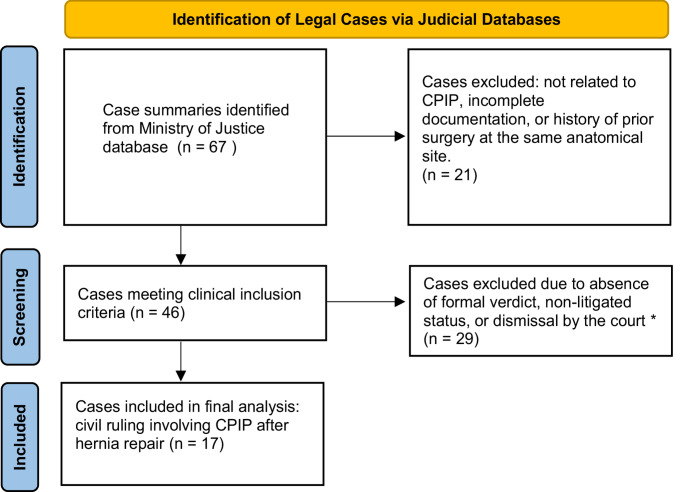
Diagram of the selection process

Eligible cases involved patients aged ≥ 18 years who underwent open or laparoscopic inguinal hernia repair and were party to a civil malpractice claim explicitly citing chronic postoperative inguinal pain. Exclusion criteria were incomplete or missing documentation, prior surgery at the same anatomical site, and concurrent unrelated abdominal procedures. Criteria were applied consistently by both reviewers to ensure uniform selection. All eligible cases were included. A detailed selection flowchart is shown in Fig. 1.

Data collection focused on descriptive reporting of litigation outcomes and related clinical variables. For each case, we recorded the compensation outcome, presence and type of nerve injury, quality of surgical documentation, surgical approach, and use of diagnostic tools such as electromyography. Clinical, procedural, and demographic data were extracted via a structured template. Missing values were excluded from specific analyses but retained in the master dataset. Collected variables included demographics (age, sex), clinical details (hernia diameter, laterality, primary vs. recurrent hernia, surgical technique—Trabucco or Lichtenstein—fixation method, treatment setting), and surgical documentation (operative reports and informed consent forms, noting mention of nerve injury risk). Additional data covered the nerve involved (ilioinguinal, iliohypogastric, genitofemoral), timing and nature of postoperative symptoms (e.g., pain, hypoesthesia), use of diagnostic tools, and any reoperations (neurectomy, orchiectomy, mesh removal). The final ruling and compensation status were also noted. Data analysis was performed using Microsoft Excel (version 11). Categorical variables are reported as absolute counts and percentages; continuous variables, as medians with interquartile ranges where applicable. No inferential statistical analyses were conducted.

## Results

A total of 67 case summaries were initially identified through the database review. After excluding records unrelated to chronic postoperative inguinal pain (CPIP), 46 met the predefined inclusion criteria, specifically involving CPIP after inguinal hernia repair. Of these, 17 cases proceeded to formal medical malpractice litigation with published judicial outcomes and were included in the final analysis (Fig. 1).

1. Patient demographics
  Of the 17 patients, 9 were male (52.9%) and 8 were female (47.1%). Compensation was awarded in 4 cases (23.5% overall), comprising 3 male patients (33.3% of all males) and 1 female patient (12.5% of all females).
2. Surgical setting and technique
  The surgical setting was specified in 8 cases (47.1%), with day surgery performed in 6 (75.0%) and inpatient hospitalization in 2 (25.0%). Compensation was awarded in 3 of the day surgery cases and in 1 of the inpatient cases..
  The surgical technique was reported in 6 cases: Trabucco (*n* = 3) and Lichtenstein (*n* = 3). Compensation was granted in 2 of the 3 Lichtenstein cases (66.7%) and in 1 of the 3 Trabucco cases (33.3%).
  The prosthetic fixation method was documented in 11 cases (64.7%). Fibrin glue was used in 1 case (9.1%), while other techniques (e.g., sutures) were more common. No compensation was awarded in any of the cases where the fixation method was documented.
3. Operative reports and informed consent
  A structured operative report was available in 6 cases (35.3%), with 1 resulting in compensation (16.7%). Informed consent documentation specifically addressing the risk of inguinal nerve injury was present in 5 cases (29.4%). Of these, 3 consents did not mention the risk (*n* = 1/3 compensated), and 2 explicitly included it (*n* = 1/2 compensated).
4. Nerve injury and symptom characteristics
  Inguinal nerve injury was documented in 13 cases (76.5%), involving:
  Ilioinguinal nerve: 7 cases (53.8%)
  Iliohypogastric nerve: 6 cases (46.2%)
  Genitofemoral nerve: 6 cases (46.2%)
  Compensation was awarded in:
  3 of 7 cases with ilioinguinal injury (42.9%)
  3 of 6 cases with iliohypogastric injury (50.0%)
  4 of 6 cases with genitofemoral injury (66.7%)
    Symptom characterization was available in 16 cases (94.1%). Inguinal pain was reported in 12 patients (75.0%), with early postoperative onset in 11 (91.7%) and delayed onset in 1 (8.3%). Hypoesthesia occurred in 4 patients (25.0%), with onset early in 2 and delayed in 2.
    Electromyography was performed in only 1 case (5.9%).
5. Surgical reinterventions
  Reintervention was documented in 5 cases (29.4%), all involving neurectomy. Three patients underwent selective neurectomy and two underwent triple neurectomy. Other procedures included mesh excision in 2 cases and orchiectomy in 1 case. Compensation was awarded in 3 of the neurectomy cases, in both mesh excision cases, and in the single orchiectomy case.
  Among the 17 analyzed cases, compensation was awarded in 3 of 5 (60.0%) neurectomy cases compared with 1 of 12 (8.3%) cases without neurectomy.
  A significant association was also observed between genitofemoral nerve injury and compensation: 4 of 6 patients (66.7%) with documented genitofemoral involvement received compensation, whereas none of the 11 patients without such injury did.
  Given the small sample size and descriptive nature of the data, these findings should be interpreted as exploratory and hypothesis-generating.

## Discussion

Italy’s national healthcare system (SSN) offers universal coverage; however, long wait times have increasingly driven patients toward private care [11]. While clinical and technological advancements have improved procedural efficacy, these developments have coincided with rising costs and growing patient dissatisfaction — reflected in the increasing volume of malpractice litigation across surgical specialties [12, 13]. In this context, CPIP-related claims remain a legally and clinically complex domain [14].

Countries such as Sweden, France, and the Netherlands have implemented extrajudicial mediation systems to manage medical disputes, while Italy is gradually adopting similar conciliatory frameworks [15, 16]. In Italian public hospitals, malpractice-related compensation is frequently handled through administrative resolutions aimed at **limiting reputational damage and legal exposure** [14]. This often leads to reimbursement agreements, followed by administrative proceedings in which physicians may be held financially accountable if state funds were misallocated [15, 16].

Given the limited number of cases (*n* = 17), this study is best classified as a retrospective case series. It provides a descriptive overview of clinical and procedural features observed in malpractice verdicts related to CPIP, reporting frequencies and distributions without testing hypotheses or evaluating associations. This approach allows for preliminary insight into the medico-legal implications of CPIP, without claiming statistical generalizability.

This study offers the first targeted analysis of medico-legal claims related to inguinal nerve injury following hernia repair in Italy. Although such complications are well-documented in surgical literature, their legal, financial, and ethical ramifications remain largely underexplored. Examining reimbursement trends alongside nerve injury characteristics and treatment modalities may help identify key medico-legal factors relevant to both surgical practice and legal frameworks. CPIP is more precisely defined as an unpleasant sensory experience—not limited to pain—in the groin area, persisting for over three months following inguinal hernia repair [17]. Although CPIP is a well-recognized clinical entity, published studies assessing its incidence and impact are unfortunately too heterogeneous to provide conclusive or generalizable results [18].

In this series, inguinal nerve injury was documented in 76.5% of cases, involving the ilioinguinal, iliohypogastric, and genitofemoral nerves in similar proportions. Inguinal pain was reported in 91.7% of cases, most of which had an early postoperative onset. These descriptive findings reflect the frequent documentation of neuropathic complications in the context of post-herniorrhaphy litigation.

A closer look at the findings shows that operative documentation was often incomplete, with fixation methods reported in only 64.7% of cases. Inadequate or missing records, while not sufficient on their own to establish liability, may nonetheless play a role in judicial reasoning, as courts can draw inferences when documentation does not allow a proper assessment of the standard of care provided [19].

In the reviewed cases, compensation was more frequently observed when genitofemoral nerve injury was reported, and in patients who underwent neurectomy. These descriptive patterns highlight elements that were recurrent in litigation outcomes, but the small sample size prevents drawing firm conclusions.

While CPIP is a recognized postoperative complication, the medico-legal dimensions of nerve injury and compensation outcomes remain largely understudied. Within the 17 cases analyzed, a predominance of documented nerve injury (76.5%) and inguinal pain (75%) reinforces ongoing concerns about post-herniorrhaphy neuropathy.

The availability of detailed surgical records—including operative reports, fixation methods, and consent forms—was frequently limited. While incomplete documentation alone does not establish liability, its absence may influence judicial reasoning, as previously reported in Italian medico-legal literature. For example, inadequate consent disclosure was noted in the majority of cases, with nerve injury risk omitted in 60% of the reviewed consent forms—despite its known relevance in hernia surgery.

It is currently unclear whether certain types of nerve injury are perceived by courts as more egregious or avoidable than others. In the reviewed cases, compensation was more frequently reported when genitofemoral nerve involvement was documented. However, this descriptive observation cannot be interpreted as evidence of judicial preference without further information on court reasoning or verdict rationale.

The low frequency of objective diagnostic testing (e.g., electromyography) is also notable, particularly given the subjective nature of CPIP-related symptoms.

The limited use of objective diagnostic testing (e.g., electromyography) is particularly noteworthy, given the subjective nature of CPIP-related symptoms. Comparative data from international jurisdictions are scarce, but reports from the UK and United States describe similar patterns, where nerve injury and testicular complications were among the most frequent causes of litigation following hernia repair [20]. In one U.S. series, nerve damage and chronic pain accounted for nearly half of malpractice claims, while orchiectomy and sexual dysfunction were frequently involved in cases that led to compensation [21].

Many of the reviewed cases involved the Trabucco or Lichtenstein techniques, as well as both day surgery and inpatient settings. Within this limited sample, compensation outcomes appeared similar across these groups. Descriptively, the type and severity of the complication emerged as more influential in legal proceedings than the surgical technique or care setting.

### Limits

This study presents several limitations that must be acknowledged.

First, the small sample size (*n* = 17) and the retrospective case series design limit the generalizability of the findings. Second, the analysis relies on judicial case summaries rather than standardized clinical records, which restricts the granularity and objectivity of the available data—particularly regarding symptom characterization, surgical technique, and diagnostic methods. Medical details may be underreported if they were not central to the litigation.

Third, the dataset includes only cases that progressed to formal litigation with published court rulings. It excludes extrajudicial settlements, administrative resolutions, and non-litigated complaints, which are common outcomes in the Italian medico-legal landscape. This introduces a selection bias toward more severe or contested cases.

Fourth, the absence of a control group (e.g., hernia patients without CPIP or without litigation) limits the ability to contextualize the findings within routine surgical practice. Without longitudinal follow-up or objective clinical validation (e.g., standardized diagnostic testing), causal inferences between surgical events and legal outcomes remain speculative.

Finally, differences in legal systems, reporting standards, and healthcare structures limit cross-national comparisons. Although foreign case series were cited for context, a systematic international analysis was beyond the scope of this study.

In summary, these limitations underscore the descriptive nature of the study and highlight the need for larger, prospective investigations to better define the medico-legal implications of CPIP.

## Conclusions and Implications

This study explores the medico-legal aspects of litigation related to chronic post-herniorrhaphy inguinal pain (CPIP) within the Italian legal system. Despite limitations related to the sample size, retrospective design, and reliance on judicial summaries, the analysis highlights recurring patterns involving nerve injury—particularly of the genitofemoral nerve—and documentation practices in malpractice claims. Within this limited series, compensation was more frequently observed in cases where genitofemoral nerve injury was reported, but these observations must be interpreted cautiously given the small cohort, absence of a control group, and lack of information on non-litigated or settled cases. These findings underscore the complexity of CPIP-related litigation and point to the need for further investigation. In particular, larger prospective studies incorporating standardized clinical data are essential to clarify the medico-legal relevance of intraoperative practices, documentation, and postoperative symptomatology. Ultimately, this study provides a descriptive overview of CPIP-related legal outcomes and aims to inform future research and support more robust medico-legal frameworks in surgical practice.
